# Cohort Study Examining the Association of Optimal Blood Pressure Control at Entry With Infrarenal Abdominal Aortic Aneurysm Growth

**DOI:** 10.3389/fcvm.2022.868889

**Published:** 2022-05-03

**Authors:** Diana Thomas Manapurathe, Joseph Vaughan Moxon, Smriti Murali Krishna, Frank Quigley, Michael Bourke, Bernard Bourke, Rhondda E. Jones, Jonathan Golledge

**Affiliations:** ^1^Queensland Research Centre for Peripheral Vascular Disease, College of Medicine and Dentistry, James Cook University, Townsville, QLD, Australia; ^2^The Australian Institute of Tropical Health and Medicine, James Cook University, Townsville, QLD, Australia; ^3^Mater Hospital, Townsville, QLD, Australia; ^4^Gosford Vascular Services, Gosford, NSW, Australia; ^5^Department of Vascular and Endovascular Surgery, The Townsville University Hospital, Townsville, QLD, Australia

**Keywords:** abdominal aortic aneurysm, systolic blood pressure, diastolic blood pressure, hypertension, AAA growth

## Abstract

**Background and Aim:**

The benefit of controlling cardiovascular risk factors in slowing the progression of small abdominal aortic aneurysm (AAA) is controversial. This study investigated the association of optimal blood pressure control at entry with the growth of small AAA.

**Methods and Results:**

A total of 1,293 patients with initial AAA diameter <50 mm were followed by a median 5 (inter-quartile range, IQR, 3–7) ultrasound scans for a median of 3.6 years (IQR 1.8, 5.3). Optimal blood pressure control was defined as blood pressure ≤140/90 mmHg at recruitment. The association of optimal blood pressure control at entry with AAA growth was assessed using linear mixed effects models adjusted for established risk factors of AAA growth and factors which were unequally distributed among the blood pressure groups. Optimal blood pressure control at entry was not significantly associated with AAA growth. In the risk factor adjusted model the mean difference in AAA growth between blood pressure groups was 0.04 mm/year (95% CI −0.20, 0.13; *p* = 0.65). The results were similar in sensitivity analyses excluding outliers or focused on systolic or diastolic blood pressure alone.

**Conclusions:**

This observational study suggests that optimal blood pressure control at entry is not associated with slower AAA growth.

## Introduction

Abdominal aortic aneurysm (AAA) is an important cause of mortality in older people (1). In many parts of the world, due to the frequent use of abdominal imaging, AAAs are identified when asymptomatic and of small diameter (2, 3). Small AAAs have a low risk of rupture, but about 50% grow to a larger size (≥55 mm), associated with a much higher risk of rupture (1, 4). There are currently no drug treatments with proven efficacy in limiting AAA growth (5).

Hypertension is an established risk factor for AAA diagnosis, however, there is lack of evidence on whether controlling blood pressure can reduce AAA growth. Hemodynamic factors, such as peak wall stress, which have been associated with AAA growth and rupture, are dependent on systemic blood pressure, suggesting that the blood pressure could be an important target to limit AAA progression (4, 6, 7). Past clinical trials testing blood pressure lowering medications have reported no effect of propranolol, perindopril, amlodipine or telmisartan on AAA growth, however, these trials were limited by small sample sizes and underpowered to identify a moderate treatment effect (8–11).

Several observational studies and meta-analyses have evaluated whether blood pressure or hypertension is associated with AAA growth (12–22). Some studies have reported hypertension was associated with an increased AAA growth rate, (12, 15, 16, 20) while others have found no association (13, 14, 17, 18, 21). These studies have used inconsistent definitions of hypertension including either history, (12, 13, 15, 17) medication prescription, (15, 20, 21) actual blood pressure measurements (13, 14, 16, 18–22) or a combination of these. This may have contributed to the inconsistent findings from these studies and it remains uncertain whether blood pressure lowering reduces AAA growth.

Current guidelines recommend maintaining blood pressure less or equal to 140/90 mmHg in people diagnosed with AAA in order to reduce the risk of cardiovascular events (23, 24). There has however been no study of whether having a blood pressure ≤140/90 mmHg is associated with slower AAA growth. The aim of this study was to assess whether people with a blood pressure measured in the out-patient clinic of ≤140/90 mmHg had slower AAA growth than those who did not meet this target.

## Patients And Methods

### Study Design and Participants

This investigation was designed as part of an ongoing prospective cohort study that aims to identify risk factors associated with AAA diagnosis and outcome. The study commenced in 2002 and remains ongoing. Patients were recruited from vascular services in Australia, including Townsville University Hospital, the Mater Hospital Townsville, Gosford Vascular Services and The Royal Brisbane and Women's Hospital. The patients were initially identified through outpatient and surveillance programs at the respective hospitals. For inclusion in the current study, patients had to have infrarenal AAA diagnosed by a vascular specialist; at least two ultrasound scans to monitor AAA growth; an initial AAA diameter <50 mm; minimum follow up of 6 months; and the assessment of blood pressure at recruitment. These inclusion criteria were selected in order to enable testing of our hypothesis that optimal blood pressure control was associated with faster AAA growth. This study included data for a follow up period of up to 6 years. Patients with a AAA diameter ≥50 mm, symptomatic AAA patients and patients who had a AAA repair were all excluded from the study. The study was performed in accordance with the Declaration of Helsinki, and ethical approval was granted from the respective institutional Ethics Committees. Written informed consent was obtained from all participants.

### Risk Factors and Medications

Risk factors, and medications of participants were recorded at study entry by clinical interview and physical examination. Smoking status was classified as ever (including current and former smoker) or never smoker (25–27). Hypertension, diabetes and stroke were defined by a history of diagnosis or treatment for these conditions (25–27). Ischemic heart disease (IHD) was defined by a history of myocardial infarction (MI), angina, or treatment for IHD (25–27). Body mass index (BMI) was measured as previously described (28). The patient's medications were recorded at recruitment, including anti-platelets, diuretics, frusemide, angiotensin converting enzyme inhibitors (ACEI), angiotensin receptor blockers (ARB), beta blockers (BB), calcium channel blockers (CCB), HMG-CoA reductase inhibitors (statins), fibrates, metformin and other hypoglycaemic agents.

### Blood Pressure

This was measured at recruitment using a digital blood pressure monitor - Omron Intellisense (HEM – 907) according to current clinical guidelines (29). Resting blood pressure was measured at the patient's first visit. Three blood pressure measurements were recorded in the right arm at 3-min intervals and the average was used. Study investigators did not undertake any blood pressure management which was under the care of the participants' general practitioners. A sub-set of patients had repeat blood pressure measurements during follow-up as part of standard clinical care.

### AAA Imaging

Maximum anterior to posterior and transverse infrarenal aortic diameters were measured by an experienced sonographer using ultrasound machines employed in the vascular laboratories at each center (Toshiba Capasee (Toshiba Medical Systems, North Ryde, New South Wales, Australia); Philips HDI 5,000 (Philips Medical Systems, Bothell, Washington, USA); GE LOGIQ 9 (GE Healthcare, Chicago, Illinois, USA); Siemens Acuson Antares™ (Siemens Healthcare, Bayswater, Victoria, Australia); Philips IU22 (Philips Medical Systems) and a standard protocol, as described previously (30–32). Aortic diameter was measured from outer wall to outer wall of the artery. The reproducibility of aortic diameter measurements was assessed in each vascular laboratory, with inter-observer reproducibility coefficients being <4 mm as previously reported (30–33).

### Statistical Analysis

This study analyzed the association between blood pressure at entry and AAA growth. The primary objective was to analyze the association between optimal blood pressure at entry and AAA growth. Optimal blood pressure was defined as that ≤140/90 mmHg measured at recruitment. Blood pressure higher than this was defined as sub-optimal. Sensitivity analyses investigated the association of optimal systolic blood pressure ( ≤140mmHg) and optimal diastolic blood pressure ( ≤90 mmHg) at entry with AAA growth. This blood pressure target was chosen in line with current guidelines (23, 34). Data were analyzed using the SPSS v 23 and R statistical software packages. The quantitative data were not normally distributed confirmed using the Shapiro Wilk test. Continuous data were presented as median and inter-quartile range (IQR). Nominal data are reported as count and percentages and were compared using Chi–squared tests. It has been proposed that a clinically important difference in AAA growth is attained only if growth is reduced by at least 30% (35). In a previous study, annual increase in AAA diameter was 1.62 ± 2.45 mm (32). To detect a 30% slower AAA growth rate in people with optimal blood pressure control, at a power of 90% and an alpha of 5%, a minimum of 1,168 participants were required. The study was therefore planned to include at least 1,200 participants. Random slope, random intercept linear mixed effects (LME) models were used to examine the association between AAA growth and blood pressure groups using unadjusted and multivariable models. Multivariate model 1 was adjusted for risk factors that have consistently been associated with AAA growth including sex, smoking, diabetes and initial AAA diameter (31). The second multivariate model included these risk factors and also those that were observed to be significantly unequally distributed among the blood pressure groups (main analysis focused on optimal blood pressure control: smoking, IHD, initial diameter, sex, diabetes, stroke, aspirin, BB, frusemide, diuretics, ACEI and statin; sensitivity analysis focused on optimal systolic blood pressure control: IHD, prior stroke, aspirin, BB and statin prescription; sensitivity analysis focused on optimal diastolic blood pressure control: IHD, prior stroke, aspirin, beta blockers, ACE-I, CCB, frusemide and statin prescription). Data for an individual at different follow up time is treated as individual observations in LME leading to 6,130 observations. Individual patients were treated as random effects in all models. Time, blood pressure group and the included covariates were included as fixed effects. The interaction of time and blood pressure group was used as the test statistic for all LME-based analyses (32, 36, 37). Model fit was assessed by visual inspection of the standardized residual distribution and q-q norm plots, suggesting the presence of potentially influential outliers. Sensitivity analyses excluding outliers (defined as data points lying >4 standard deviations away from the mean of all model residuals) were performed. These were calculated using the R nlme and car packages (http://www.r-project.org), as described previously (37). *P*-values < *0.05* were considered significant for all the analyses.

## Results

### Blood Pressure Control at Recruitment and Follow-Up

Out of the 1,293 participants, 475 (36.7%) had optimum blood pressure control and 818 (63.3%) had sub-optimal blood pressure control at recruitment. Participants with optimal blood pressure control had a significantly higher prevalence of diabetes, IHD, past smoking and past stroke and they were also significantly more likely to be prescribed aspirin, frusemide, BB, ACEI, diuretics, statins and drugs to treat diabetes (Table 1). They also had significantly larger AAAs at the time of recruitment (Table 1). A sub-set of participants (*n* = 304; 23.5%) had a repeat blood pressure measurement a median (IQR) of 2.0 (1.0, 3.9) years after recruitment. Participants with sub-optimal blood pressure at recruitment had significantly higher median (IQR) systolic blood pressure when assessed during follow-up than those with optimal blood pressure at recruitment (141, 130 to 154, vs. 130, 120 to 142 mmHg; *p* < 0.001).

**Table 1:** Characteristics of the participants in relation to whether they had optimal or sub-optimal blood pressure control.

**Demographic** **and clinical** **characteristics**	**Blood pressure (BP) (*****n*** **=** **1,293)**		***P*-value**
	**Optimal BP ≤140/90 mmHg**	**Sub-optimal BP SBP >140 or DBP >90 mmHg**	
*N*	475	818	
Age (years)	73.3 (68.7–77.7)	73.4 (69.6–77.5)	0.23
Initial AAA diameter (mm)	37.0 (33.0–42.0)	34.6 (31.1–40.0)	<0.001
**Sex**			<0.001
Male	404 (85.1%)	773 (94.5%)	
Female	71 (14.9%)	45 (5.5%)	
BMI (kg/m^2^)	27.0 (25.0–30.0)	27.0 (25.0–30.0)	0.95
**Smoking**			<0.001
*Never*	105 (22.1%)	338 (41.3%)	
*Ever*	370 (77.9%)	480 (58.7%)	
eGFR	68.5 (54.6–82.5)	68.7 (55.9–81.3)	0.94
DM	100 (21.1%)	122 (14.9%)	<0.01
Hypertension	320 (67.4%)	513 (62.7%)	0.09
IHD	249 (52.4%)	325 (39.8%)	<0.001
Stroke	50 (10.5%)	56 (6.8%)	0.02
**Medications**			
Aspirin	261 (54.9%)	305 (37.3%)	<0.001
Other antiplatelets	55 (11.6%)	74 (9.0%)	0.14
CCB	87 (18.3%)	128 (15.6%)	0.21
Frusemide	45 (9.5%)	49 (6.0%)	0.02
Beta blocker	147 (30.9%)	172 (21.0%)	<0.001
ACE I	152 (32.0%)	204 (24.9%)	<0.01
ARB	78 (16.4%)	111 (13.6%)	0.16
Diuretics	48 (10.1%)	49 (6.0%)	<0.01
Statins	279 (58.7%)	329 (40.2%)	<0.001
Fibrates	12 (2.5%)	5 (0.6%)	<0.01
Metformin	48 (10.1%)	57 (7.0%)	0.05
Other hypoglycemic agents	39 (8.2%)	41 (5.0%)	0.02
Follow-up (years)	2.8 (1.2–5.0)	4.0 (2.0–5.6)	<0.001

*The data were expressed as median (IQR) for continuous data and n (%) for categorical data. AAA, Abdominal aortic aneurysm; ACE I, angiotensin converting enzyme inhibitor; ARB, angiotensin receptor blockers; BMI, Body mass index; CCB, Calcium channel blocker, CHD, coronary heart disease; DBP, diastolic blood pressure, DM, Diabetes mellitus; SBP, systolic blood pressure; Missing data: BMI - 15, eGFR - 393*.

### Association of Optimal Blood Pressure Control and AAA Growth

**Figure 1:**
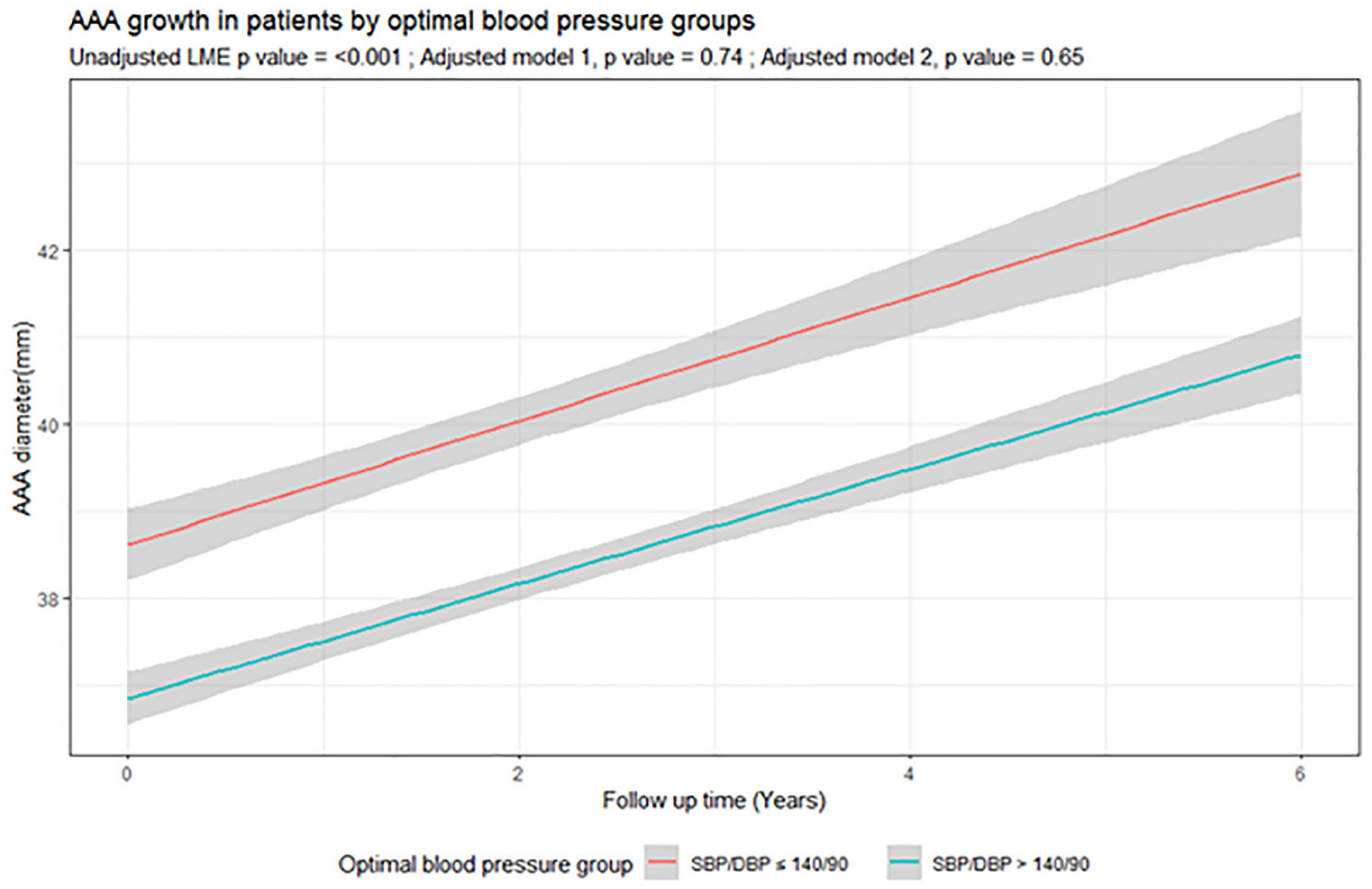
Association of optimal blood pressure with abdominal aortic aneurysm growth. The graph illustrates the mean growth (95% CI) of AAA growth during follow up (years) according to optimal blood pressure in AAA patients. The red line represents patients with BP ≤140/90 mmHg and the green line represents patients with SBP >140 or DBP >90 mmHg. BP, blood pressure; DBP, diastolic blood pressure; SBP, systolic blood pressure.

Participants were followed by a median of 5 (inter-quartile range, IQR, 3, 7) ultrasound scans for a median of 3.6 years (IQR 1.8, 5.3). The unadjusted analysis suggested that participants with sub-optimal blood pressure control had significantly slower AAA growth. The mean difference in AAA growth between blood pressure groups was 1.75 mm/year (95% CI 2.37, 1.12) *p* < 0.001 (Table 2, Figure 1). This association did not remain significant when adjusted for other risk factors (Model 1: The mean difference of AAA growth was −0.03 mm/year (95% CI −0.19, 0.13, *p* = 0.74; Model 2: The mean difference of AAA growth was −0.04 mm/year (95% CI −0.20, 0.13, *p* = 0.65) (Table 2, Supplementary 1 Table 1). A sensitivity analysis excluding potential outliers did not alter the findings (Table 3, Supplementary 1 Table 2).

**Table 2:** Association between optimal blood pressure control and AAA growth.

	**Number of participants** **(n = 1,293, number of observations = 6,130)**	**Mean difference in AAA growth per year**	**95% CI**	**P-value**
Unadjusted model	Optimal	Reference		
	Sub-optimal	−1.75	−2.37 to −1.12	<0.001
Adjusted model 1	Optimal	Reference		
	Sub-optimal	−0.03	−0.19–0.13	0.74
Adjusted model 2	Optimal	Reference		
	Sub-optimal	−0.04	−0.20–0.13	0.65

*Optimal BP – SBP/DBP ≤140/90 mmHg and sub-optimal BP – SBP >140 or DBP >90 mmHg. Model 1 was adjusted for smoking, DM, initial diameter and sex and Model 2 was adjusted for smoking, IHD, initial diameter, sex, DM, stroke, aspirin, BB, frusemide, diuretics, ACEI and statin. Cited p-values ≈ β = interaction of time and blood pressure groups. ACEI, angiotensin converting enzyme inhibitor; BB, beta blocker; BP, blood pressure; CI, confidence interval; DBP, diastolic blood pressure; DM, diabetes mellitus; IHD, ischemic heart disease; N, sample size; SBP, systolic blood pressure. The full model is elaborated in Supplementary 1 Table 1*.

**Table 3:** Association between optimal blood pressure and AAA growth after removing participants with outlier measurements.

	**Number of participants** **(*n* = 1,293, number of observations = 6,005)**	**Mean difference in AAA growth per year**	**95% CI**	***P*-value**
Unadjusted model	Optimal	Reference		
	Sub-optimal	−1.74	−2.37 to −1.12	<0.001
Adjusted model 1	Optimal	Reference		
	Sub-optimal	0.01	−0.10–0.13	0.81
Adjusted model 2	Optimal	Reference		
	Sub-optimal	0.001	−0.12–0.12	0.99

*Optimal BP – SBP/DBP ≤140/90 mmHg and sub-optimal BP – SBP >140 or DBP >90 mmHg. Model 1 was adjusted for smoking, DM, initial diameter and sex and Model 2 was adjusted for smoking, IHD, initial diameter, sex, DM, stroke, aspirin, BB, frusemide, diuretics, ACEI and statin. Cited p-values ≈ β = interaction of time and blood pressure groups. ACEI, angiotensin converting enzyme inhibitor; BB, beta blocker; BP, blood pressure; CI, confidence interval; DBP, diastolic blood pressure; DM, diabetes mellitus; IHD, ischemic heart disease; N, sample size; SBP, systolic blood pressure. The full model is elaborated in Supplementary 1 Table 2*.

### Association of Optimal Systolic Blood Pressure Control With AAA Growth

At recruitment, 503 (38.9%) participants had systolic blood pressure ≤140 mm Hg and 790 (61.6%) had systolic blood pressure >140 mmHg (Supplementary 2 Table 1). The association of systolic blood pressure groups with risk factors is shown in Supplementary 2 Table 2. AAA growth was significantly slower in patients with systolic blood pressure > than ≤140 mmHg in the unadjusted analysis. The mean difference in AAA growth was 1.53 mm/year (95% CI −2.15, −0.91; *p* <0.001; Supplementary 2 Table 2). This association did not remain significant when adjusted for other risk factors (Model 1: mean difference in AAA growth was −0.0003, 95% CI −0.16, 0.16, *p* = 1.00; Model 2: mean difference in AAA growth was −0.01, 95% CI −0.17, 0.15, *p* = 0.90). A sensitivity analysis excluding potential outliers did not alter the findings (Supplementary 2 Table 3). Furthermore, systolic blood pressure recorded at entry as a continuous variable was not significantly associated with AAA growth after adjusting for other risk factors (Supplementary 2 Table 4).

### Association of Diastolic Blood Pressure With AAA Growth

At recruitment, 917 (67.3%) participants had a diastolic blood pressure ≤90 mmHg and 376 (27.6%) had a diastolic blood pressure >90 mmHg. The association of diastolic blood pressure groups with risk factors is shown in Supplementary 3 Table 1. Similar to other analyses AAA growth was significantly slower in patients with diastolic blood pressure > than ≤90 mmHg in unadjusted (mean difference in AAA growth was −1.78 mm/year, 95% CI −2.45, −1.12, *p* = <0.001, Supplementary 3 Table 2) but not adjusted analyses (Model 1: mean difference in AAA growth was−0.14, 95% CI−0.32, 0.02, p = 0.09; Model 2: mean difference in AAA growth was −0.16, 95% CI −0.33, 0.01, *p* = 0.07). Removal of potentially influential outliers did not change these results (Supplementary 3 Table 3). Additional analyses using diastolic blood pressure measured at entry as a continuous variable show no significant association with AAA growth after adjusting for other risk factors (Supplementary 3 Table 4).

## Discussion

The findings of the current study suggest that guideline recommended control of blood pressure at entry is not associated with reduced AAA growth in patients that have small AAA. Findings were consistent in sensitivity analyses. The unadjusted analysis suggested that patients with suboptimal blood pressure at entry had reduced AAA growth. This association was however not maintained in analyses adjusted for importance confounding factors. Larger initial AAA diameter was the major predictor of faster AAA growth. However, no interaction was observed between the initial diameter and AAA growth, suggesting that blood pressure levels does not affect AAA growth depending on the initial diameter.

Several observational studies and meta-analyses have evaluated whether blood pressure or hypertension is associated with AAA growth (12–22). Overall these studies have suggested an inconsitent association of blood pressure with AAA growth, in keeping with the findings of the current study.

A number of small randomized control trials have assessed the effect of blood pressure lowering medications, including propranolol, amlodipine, perindopril and telmisartan, on AAA growth (8–11). None of these studies found that these anti-hypertensive medications slowed AAA growth. All these studies were underpowered, but a meta-analysis of these trials suggested that blood pressure lowering did not slow AAA growth or reduce requirement for AAA repair (38, 39).

Due to the small size of these prior randomized trials and the limited range of anti-hypertensive drugs tested, it remains uncertain whether blood pressure lowering reduces AAA growth. Observational studies may provide some evidence whether high blood pressure is a treatment target to limit AAA growth, although in previous such studies findings have been inconsistent (12–14, 16, 17, 20–22). A previous meta-analysis reported that hypertension was not associated with AAA growth although it is notable that the definitions of hypertension, samples sizes and methods of examining AAA growth varied which may have contributed to the inconsistency reported (40). The current study represents the largest cohort using a clear definition of optimal blood pressure control and standard method of measuring AAA diameter to investigate the association of blood pressure with AAA growth. The findings suggest that optimal blood pressure control does not slow AAA growth. It is well-established that hypertension is an important risk factors for cardiovascular events, such as MI and stroke, which are common in people with AAA and therefore controlling blood pressure in patients with small AAA remains important despite the lack of evidence on its effect on AAA growth (40, 41).

The current study has a number of strengths and limitations. In comparison to the other observational studies, the current study had a large sample size in which data were collected according to standardized protocols. This allowed for well-powered analyses. Also, a range of sensitivity and adjusted analyses were performed. The main limitation of the study was that the participants only had one blood pressure measurement performed at study entry. Only patients with clinical requirement had repeat blood pressure measurements and a separate analysis could not be performed due to the small sample size. Medical management changes were also not recorded during the study. Blood pressure was only measured in the right arm which could have resulted in underestimation of values in the presence of subclavian artery stenosis or occlusion. Even though the study included a large number of patients, there was a difference in sample sizes of the two blood pressure groups which may have influenced the power of the analyses. The current study was also not a randomized control trial; hence it remains possible that factors we did not measure including chronic airways disease and therefore could not adjust for led to residual confounding. AAA diameter was measured with ultrasound rather than computed tomography but we have previously reported excellent reproducibility of ultrasound measured AAA diameter (30–33).

In conclusion this study suggests that optimal control of blood pressure at entry is unlikely to limit growth of small AAA. This does not outweigh the benefits of blood pressure management in AAA patients to reduce cardiovascular events and other AAA complications. Further well-designed studies including patients with multiple blood pressure measurements are required for conclusive evidence.

## Data Availability Statement

The original contributions presented in the study are included in the article/Supplementary Material, further inquiries can be directed to the corresponding author/s.

## Ethics Statement

The studies involving human participants were reviewed and approved by the Townsville Hospital and Health Services Ethics Committee. The patients/participants provided their written informed consent to participate in this study.

## Author Contributions

DT was involved in project administration, data curation, conceptualization, formal analysis, investigation, methodology, validation, visualization, writing, and editing. JG was involved in project administration, conceptualization, funding acquisition, data collection, investigation, methodology, supervision, writing, and editing. JM was involved in conceptualization, formal analysis, investigation, methodology, supervision, validation, and editing. SK was involved in supervision and editing. FQ, MB, and BB were involved in data collection and data curation and editing. RJ was involved in formal analysis, methodology, and editing. All authors contributed to the article and approved the submitted version.

## Funding

This study was supported by funding from the National Health and Medical Research Council (1063476 and 1180736) and the Queensland Government (Australia). JG holds a Practitioner Fellowship from the National Health and Medical Research Council (1117061) and a Senior Clinical Research Fellowship from the Queensland Government. JM was supported by an Advance Queensland Fellowship from the Queensland Government. DT was supported by a JCU (James Cook University) Postgraduate Research Scholarship and a JCU College of Medicine and Dentistry Scholarship.

## Conflict of Interest

The authors declare that the research was conducted in the absence of any commercial or financial relationships that could be construed as a potential conflict of interest.

## Publisher's Note

All claims expressed in this article are solely those of the authors and do not necessarily represent those of their affiliated organizations, or those of the publisher, the editors and the reviewers. Any product that may be evaluated in this article, or claim that may be made by its manufacturer, is not guaranteed or endorsed by the publisher.
