# Supplementary material for: Cohort Study Examining the Association of Optimal Blood Pressure Control at Entry With Infrarenal Abdominal Aortic Aneurysm Growth
Source: Front Cardiovasc Med. 2022 May 3;9:868889. doi: 10.3389/fcvm.2022.868889 (PMC9110652; doi:10.3389/fcvm.2022.868889)
Supplement: Supplementary file 1 [file Data_Sheet_1.docx]

# Supplemental 1

**Table 1 – Association of optimal BP and AAA growth**

|  | **Number of participants (n = 1293,**  **number of observations =**  **6130)** | **Mean difference in AAA growth per year** | **95 % CI** | **P value** |
| --- | --- | --- | --- | --- |
| Unadjusted model | Optimal | REF |  |  |
|  | Sub-optimal | -1.75 | -2.37 – -1.12 | <0.001 |
| Adjusted model 1 | Optimal | REF |  |  |
|  | Sub-optimal | -0.03 | -0.19 – 0.13 | 0.74 |
|  | Initial diameter | 0.98 | 0.96 – 0.99 | <0.001 |
|  | Smoking | 0.08 | -0.08 – 0.24 | 0.35 |
|  | DM | -0.11 | -0.32 – 0.09 | 0.27 |
|  | Sex | -0.13 | -0.40 – 0.13 | 0.33 |
| Adjusted model 2 | Optimal | REF |  |  |
|  | Sub-optimal | -0.04 | -0.20 – 0.13 | 0.65 |
|  | Initial diameter | 0.98 | 0.97 – 0.99 | <0.001 |
|  | Smoking | 0.08 | -0.09 – 0.26 | 0.33 |
|  | DM | -0.09 | -0.30 – 0.11 | 0.37 |
|  | Sex | -0.11 | -0.39 – 0.16 | 0.41 |
|  | Stroke | -0.04 | -0.32 – 0.24 | 0.78 |

|  | Frusemide | -0.17 | -0.46 – 0.13 | 0.27 |
| --- | --- | --- | --- | --- |
|  | Diuretic | -0.18 | -0.48 – 0.11 | 0.23 |
|  | Aspirin | -0.12 | -0.29 – 0.06 | 0.19 |
|  | IHD | 0.05 | -0.11 – 0.21 | 0.56 |
|  | Beta blocker | 0.07 | -0.13 – 0.26 | 0.50 |
|  | statin | 0.004 | -0.18 – 0.19 | 0.96 |
|  | ACEI | 0.04 | -0.13 – 0.23 | 0.62 |

Optimal BP – SBP/DBP ≤ 140/90 mmHg and sub-optimal BP – SBP > 140 or DBP > 90 mmHg. Model 1 was adjusted for smoking, DM, initial diameter and sex and Model 2 was adjusted for smoking, IHD, initial diameter, sex, DM, stroke, aspirin, BB, frusemide, diuretics, ACEI and statin. Cited p values ≈ β = interaction of time and blood pressure groups. Abbreviations: AAA – abdominal aortic aneurysm, ACEI – angiotensin converting enzyme inhibitor, BB – beta blocker, BP – blood pressure, CI – confidence interval, DBP – diastolic blood pressure, DM – diabetes mellitus, IHD – ischemic heart disease, N – sample size, SBP – systolic blood pressure.

# Table 2 – Association between optimal BP and AAA growth after removing outliers

|  | **Number of participants**  **(n = 1293, number**  **of observations = 6005)** | **Mean difference in AAA growth per year** | **95 % CI** | **P value** |
| --- | --- | --- | --- | --- |
| Unadjusted model | Optimal | REF |  |  |
|  | Sub-optimal | -1.74 | -2.37 – -1.12 | <0.001 |
| Adjusted model 1 | Optimal | REF |  |  |
|  | Sub-optimal | 0.01 | -0.10 – 0.13 | 0.81 |
|  | Initial diameter | 0.99 | 0.98 – 1.00 | <0.001 |
|  | Smoking | 0.07 | -0.05 – 0.18 | 0.26 |
|  | DM | -0.05 | -0.19 – 0.10 | 0.53 |
|  | Sex | -0.14 | -0.34 – 0.05 | 0.16 |
| Adjusted model 2 | Optimal | REF |  |  |
|  | Sub-optimal | 0.001 | -0.12 – 0.12 | 0.99 |
|  | Initial diameter | 0.99 | 0.98 – 1.00 | <0.001 |
|  | Smoking | 0.08 | -0.04 – 0.21 | 0.21 |
|  | DM | -0.04 | -0.19 – 0.11 | 0.57 |
|  | Sex | -0.13 | -0.33 – 0.07 | 0.20 |
|  | Stroke | 0.04 | -0.17 – 0.24 | 0.72 |
|  | Frusemide | -0.17 | -0.39 – 0.04 | 0.11 |
|  | Diuretic | 0.03 | -0.19 – 0.25 | 0.78 |
|  | Aspirin | -0.10 | -0.23 – 0.02 | 0.11 |
|  | IHD | 0.002 | -0.11 – 0.12 | 0.96 |
|  | Beta blocker | -0.03 | -0.18 – 0.11 | 0.63 |
|  | statin | 0.004 | -0.13 – 0.14 | 0.96 |
|  | ACEI | 0.06 | -0.07 – 0.19 | 0.37 |

Optimal BP – SBP/DBP ≤ 140/90 mmHg and sub-optimal BP – SBP > 140 or DBP > 90 mmHg. Model 1 was adjusted for smoking, DM, initial diameter and sex and Model 2 was adjusted for smoking, IHD, initial diameter, sex, DM, stroke, aspirin, BB, frusemide, diuretics, ACEI and statin. Cited p values ≈ β = interaction of time and blood pressure groups. Abbreviations: AAA – abdominal aortic aneurysm, ACEI – angiotensin converting enzyme inhibitor, BB – beta blocker, BP – blood pressure, CI – confidence interval, DBP – diastolic blood pressure, DM – diabetes mellitus, IHD – ischemic heart disease, N – sample size, SBP – systolic blood pressure.
