# Supplementary material for: Cohort Study Examining the Association of Optimal Blood Pressure Control at Entry With Infrarenal Abdominal Aortic Aneurysm Growth
Source: Front Cardiovasc Med. 2022 May 3;9:868889. doi: 10.3389/fcvm.2022.868889 (PMC9110652; doi:10.3389/fcvm.2022.868889)
Supplement: Supplementary file 2 [file Data_Sheet_2.docx]

**Supplemental 2**

**Table 1: Characteristics of the participants in relation to whether they had high or low systolic blood pressure.**

| **Demographic and clinical characteristics** | **n = 1293** | |  |
| --- | --- | --- | --- |
|  | **SBP ≤ 140 mmHg** | **SBP >140 mmHg** | **P value** |
| N | 503 | 790 |  |
| Age (years) | 73.4 (68.6 – 77.7) | 73.4 (69.6 – 77.5) | 0.30 |
| Initial AAA diameter (mm) | 36.8 (32.5 – 42.0) | 34.6 (31.2 – 40.0) | <0.001 |
| Sex | | | <0.001 |
| Male | 431 (85.7%) | 746 (94.4%) |  |
| Female | 72 (14.3%) | 44 (5.6%) |  |
| BMI (kg/m2) | 27.0 (25.0 – 30.0) | 27.0 (25.0 – 30.0) | 0.91 |
| Smoking | | | <0.001 |
| *Never* | 122 (24.3%) | 321 (40.6%) |  |
| *Ever* | 381 (75.7%) | 469 (59.4%) |  |
| DM | 103 (20.5%) | 119 (15.1%) | 0.01 |
| Hypertension | 333 (66.2%) | 500 (63.3%) | 0.29 |
| IHD | 262 (52.1%) | 312 (39.5%) | <0.001 |

| Stroke | 54 (10.7%) | 52 (6.6%) | <0.01 |
| --- | --- | --- | --- |
| Medications | | | |
| Aspirin | 270 (53.7%) | 296 (37.5%) | <0.001 |
| Other antiplatelets | 57 (11.3%) | 72 (9.1%) | 0.19 |
| CCB | 91 (18.1%) | 124 (15.7%) | 0.26 |
| Frusemide | 48 (9.5%) | 46 (5.8%) | 0.01 |
| Beta blocker | 154 (30.6%) | 165 (20.9%) | <0.001 |
| ACE I | 158 (31.4%) | 198 (25.1%) | 0.01 |
| ARB | 79 (15.7%) | 110 (13.9%) | 0.38 |
| Diuretics | 50 (9.9%) | 47 (5.9%) | 0.01 |
| Statins | 287 (57.1%) | 321 (40.6%) | <0.001 |
| Fibrates | 12 (2.4%) | 5 (0.6%) | 0.01 |
| Metformin | 51 (10.1%) | 54 (6.8%) | 0.03 |
| Other hypoglycemic agents | 39 (7.8%) | 41 (5.2%) | 0.06 |
| Follow-up (years) | 2.8 (1.3 – 5.0) | 4.0 (2.0 – 5.6) | <0.001 |

The data were expressed as median (IQR) for continuous data and n (%) for categorical data. Abbreviations: AAA – Abdominal aortic aneurysm; ACE I – angiotensin converting enzyme inhibitor; ARB – angiotensin receptor blockers; BMI- Body mass index; CCB – Calcium channel blocker; DM: Diabetes mellitus; IHD – Ischemic heart disease, IQR – interquartile range, SBP – systolic blood pressure. Missing data: BMI - 15

# Table 2 – Association between SBP and AAA growth

|  | **Number of participants (n = 1293,**  **number of**  **observations = 6130)** | **Mean difference in AAA growth per year** | **95 % CI** | **P value** |
| --- | --- | --- | --- | --- |
| Unadjusted model | SBP ≤ 140 | REF |  |  |
|  | SBP > 140 | -1.53 | -2.15 – -0.91 | <0.001 |
| Adjusted model 1 | SBP ≤ 140 | REF |  |  |
|  | SBP > 140 | -0.0003 | -0.16 – 0.16 | 1.00 |
|  | Initial diameter | 0.98 | 0.96 – 0.99 | <0.001 |
|  | Smoking | 0.08 | -0.08 – 0.24 | 0.32 |
|  | DM | -0.11 | -0.31 – 0.09 | 0.27 |
|  | Sex | -0.13 | -0.40 – 0.14 | 0.35 |
| Adjusted model 2 | SBP ≤ 140 | REF |  |  |
|  | SBP > 140 | -0.01 | -0.17 – 0.15 | 0.90 |
|  | Initial diameter | 0.98 | 0.97 – 0.99 | <0.001 |
|  | Smoking | 0.09 | -0.08 – 0.26 | 0.31 |
|  | DM | -0.09 | -0.30 – 0.11 | 0.38 |
|  | Sex | -0.11 | -0.38 – 0.16 | 0.43 |

|  | Stroke | -0.04 | -0.32 – 0.24 | 0.79 |
| --- | --- | --- | --- | --- |
|  | Frusemide | -0.17 | -0.46 – 0.13 | 0.27 |
|  | Diuretic | -0.18 | -0.48 – 0.11 | 0.23 |
|  | Aspirin | -0.11 | -0.29 – 0.06 | 0.20 |
|  | IHD | 0.05 | -0.11 – 0.21 | 0.55 |
|  | Beta blocker | 0.07 | -0.13 – 0.26 | 0.50 |
|  | statin | 0.006 | -0.18 – 0.19 | 0.95 |
|  | ACEI | 0.04 | -0.14 – 0.23 | 0.62 |

Model 1 was adjusted for smoking, DM, initial diameter and sex and Model 2 was adjusted for smoking, IHD, initial diameter, sex, DM, stroke, aspirin, BB, frusemide, diuretics, ACEI and statin. Cited p values ≈ β = interaction of time and blood pressure groups. Abbreviations : AAA – Abdominal aortic aneurysm, ACEI – angiotensin converting enzyme inhibitor, BB – beta blocker, CI – confidence interval, DM – diabetes mellitus, IHD – ischemic heart disease, N – sample size, SBP – systolic blood pressure.

# Table 3 – Association between SBP and AAA growth after removing outliers

|  | **Number of participants**  **(n = 1293, number of observations =**  **6004)** | **Mean difference in AAA growth per year** | **95 % CI** | **P value** |
| --- | --- | --- | --- | --- |
| Unadjusted model | SBP ≤ 140 | REF |  |  |
|  | SBP > 140 | -1.53 | -2.14 – -0.91 | <0.001 |
| Adjusted model 1 | SBP ≤ 140 | REF |  |  |
|  | SBP > 140 | 0.03 | -0.08 – 0.15 | 0.55 |
|  | Initial diameter | 0.99 | 0.98 – 1.00 | <0.001 |
|  | Smoking | 0.07 | -0.05 – 0.19 | 0.24 |
|  | DM | -0.05 | -0.19 – 0.10 | 0.54 |
|  | Sex | -0.14 | -0.33 – 0.06 | 0.17 |
| Adjusted model 2 | SBP ≤ 140 | REF |  |  |
|  | SBP > 140 | 0.02 | -0.09 – 0.14 | 0.72 |
|  | Initial diameter | 0.99 | 0.98 – 1.00 | <0.001 |
|  | Smoking | 0.08 | -0.04 – 0.21 | 0.19 |
|  | DM | -0.04 | -0.19 – 0.11 | 0.58 |
|  | Sex | -0.12 | -0.32 – 0.07 | 0.22 |
|  | Stroke | 0.04 | -0.16 – 0.24 | 0.71 |
|  | Frusemide | -0.17 | -0.39 – 0.04 | 0.12 |
|  | Diuretic | 0.03 | -0.19 – 0.25 | 0.78 |
|  | Aspirin | -0.10 | -0.23 – 0.02 | 0.12 |
|  | IHD | 0.003 | -0.11 – 0.12 | 0.95 |
|  | Beta blocker | -0.03 | -0.18 – 0.11 | 0.63 |
|  | statin | 0.004 | -0.13 – 0.14 | 0.95 |
|  | ACEI | 0.06 | -0.07 – 0.19 | 0.38 |

Model 1 was adjusted for smoking, DM, initial diameter and sex and Model 2 was adjusted for smoking, IHD, initial diameter, sex, DM, stroke, aspirin, BB, frusemide, diuretics, ACEI and statin. Cited p values ≈ β = interaction of time and blood pressure groups. Abbreviations: AAA- abdominal aortic aneurysm, ACEI – angiotensin converting enzyme inhibitor, BB –beta blocker, CI – confidence interval, DM – diabetes mellitus, IHD – ischemic heart disease, N – sample size, SBP – systolic blood pressure.

#
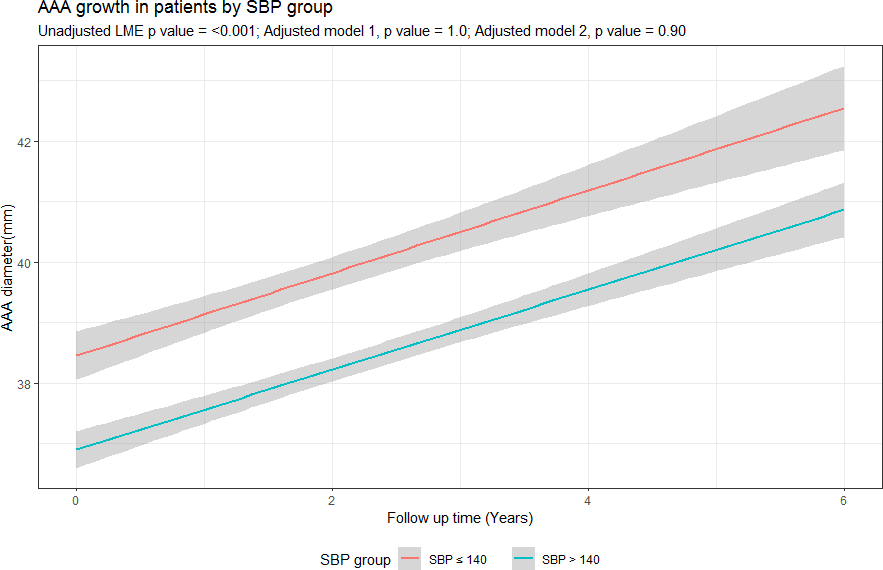
Figure 1: Association of systolic blood pressure with abdominal aortic aneurysm growth.

The graph illustrates the mean (95% CI) AAA growth during follow up (years) according to systolic blood pressure (SBP). The red line represents patients with SBP ≤ 140 mmHg and the green line represents patients with SBP >140 mmHg.

**Table 4 – Association between SBP (continuous variable) and AAA growth**

|  | **Number of participants (n = 1293, number of**  **observations = 6130)** | **Mean difference in AAA growth per year** | **95 % CI** | **P value** |
| --- | --- | --- | --- | --- |
| Unadjusted model | SBP | -0.04 | -0.05 – -0.03 | <0.001 |
| Adjusted model 1 | SBP | <0.001 | -0.003 – 0.004 | 0.65 |
| Adjusted model 2 | SBP | <0.001 | -0.003 – 0.004 | 0.77 |
| **After removing outliers** | | | | |
| Unadjusted model | SBP | -0.04 | -0.06 - -0.03 | <0.001 |
| Adjusted model 1 | SBP | <0.001 | -0.002 – 0.003 | 0.47 |
| Adjusted model 2 | SBP | <0.001 | -0.002 – 0.003 | 0.60 |

Model 1 was adjusted for smoking, DM, initial diameter and sex and Model 2 was adjusted for smoking, IHD, initial diameter, sex, DM, stroke, aspirin, BB, frusemide, diuretics, ACEI and statin. Cited p values ≈ β = interaction of time and blood pressure groups. Abbreviations: AAA- abdominal aortic aneurysm, ACEI – angiotensin converting enzyme inhibitor, BB –beta blocker, CI – confidence interval, DM – diabetes mellitus, IHD – ischemic heart disease, N – sample size, SBP – systolic blood pressure.
