# Supplementary material for: Cohort Study Examining the Association of Optimal Blood Pressure Control at Entry With Infrarenal Abdominal Aortic Aneurysm Growth
Source: Front Cardiovasc Med. 2022 May 3;9:868889. doi: 10.3389/fcvm.2022.868889 (PMC9110652; doi:10.3389/fcvm.2022.868889)
Supplement: Supplementary file 3 [file Data_Sheet_3.docx]

# Supplemental 3

**Table 1: Demographic and baseline characteristics of the AAA patients according to diastolic blood pressure**

| **Sl no** | **Demographic and clinical characteristics** | **n = 1293** | |  |
| --- | --- | --- | --- | --- |
|  |  | **DBP ≤ 90 mmHg** | **DBP > 90 mmHg** | **P value** |
| 1 | N | 917 | 376 |  |
| 2 | Age | 73.8 (69.6 – 78.0) | 72.3 (68.9 – 76.7) | 0.02 |
| 3 | Initial AAA diameter | 36.0 (32.0 – 41.6) | 33.7 (31.0 – 38.7) | <0.001 |
| 5 | Sex | | | <0.001 |
|  | Male | 810 (88.3%) | 367 (97.6%) |  |
|  | Female | 107 (11.7%) | 9 (2.4%) |  |
| 6 | BMI | 27.0 (25.0 – 30.0) | 27.0 (25.0 – 30.0) | 0.76 |
| 7 | Smoking | | | <0.001 |
|  | *Never* | 242 (26.4%) | 201 (53.5%) |  |
|  | *Ever* | 675 (73.6%) | 175 (46.5%) |  |
| 8 | DM | 181 (19.7%) | 41 (10.9%) | <0.001 |
| 9 | Hypertension | 611 (66.6%) | 222 (59.0%) | 0.01 |
| 10 | IHD | 457 (49.8%) | 117 (31.1%) | <0.001 |
| 11 | Stroke | 81 (8.8%) | 25 (6.6%) | 0.20 |

| 12 | Medications | | | |
| --- | --- | --- | --- | --- |
|  | Aspirin | 466 (50.8%) | 100 (26.6%) | <0.001 |
|  | Other antiplatelets | 100 (10.9%) | 29 (7.7%) | 0.08 |
|  | CCB | 173 (18.9%) | 42 (11.2%) | 0.001 |
|  | Frusemide | 75 (8.2%) | 19 (5.1%) | 0.05 |
|  | Beta blocker | 260 (28.4%) | 59 (15.7%) | <0.001 |
|  | ACE I | 278 (30.3%) | 78 (20.7%) | <0.001 |
|  | ARB | 143 (15.6%) | 46 (12.2%) | 0.12 |
|  | Diuretics | 81 (8.8%) | 16 (4.3%) | 0.005 |
|  | Statins | 494 (53.9%) | 114 (30.3%) | <0.001 |
|  | Fibrates | 17 (1.9%) | 0 | 0.008 |
|  | Metformin | 88 (9.6%) | 17 (4.5%) | 0.002 |
|  | Other hypoglycemic agents | 65 (7.1%) | 15 (4.0%) | 0.04 |
| 13 | Follow-up (years) | 3.1 (1.5 – 5.1) | 4.4 (2.0 – 5.7) | <0.001 |

The data are expressed as median (IQR) for continuous data and n (%) for categorical data. Abbreviations: AAA – Abdominal aortic aneurysm, ACE I – angiotensin converting enzyme inhibitor; ARB – angiotensin receptor blockers, BMI- Body mass index; CCB – Calcium channel blocker, DBP – diastolic blood pressure, DM: Diabetes mellitus, IHD – Ischemic heart disease, IQR -interquartile range. Missing data: BMI - 15

# Table 2 – Association between DBP and AAA growth

|  | **Number of participants (n = 1293,**  **number of observations = 6130)** | **Mean difference in AAA growth per year** | **95 % CI** | **P value** |
| --- | --- | --- | --- | --- |
| Unadjusted model | DBP ≤ 90 | REF |  |  |
|  | DBP > 90 | -1.78 | -2.45 – -1.12 | <0.001 |
| Adjusted model 1 | DBP ≤ 90 | REF |  |  |
|  | DBP > 90 | -0.14 | -0.32 – 0.02 | 0.09 |
|  | Initial diameter | 0.98 | 0.96 – 0.99 | <0.001 |
|  | Smoking | 0.05 | -0.11 – 0.21 | 0.54 |
|  | DM | -0.12 | -0.33 – 0.08 | 0.23 |
|  | Sex | -0.16 | -0.42 – 0.11 | 0.26 |
| Adjusted model 2 | DBP ≤ 90 | REF |  |  |
|  | DBP > 90 | -0.16 | -0.33 – 0.01 | 0.07 |
|  | Initial diameter | 0.98 | 0.96 – 0.99 | <0.001 |
|  | Smoking | 0.06 | -0.11 – 0.23 | 0.50 |
|  | DM | -0.12 | -0.32 – 0.09 | 0.26 |

|  | Sex | -0.14 | -0.41 – 0.13 | 0.29 |
| --- | --- | --- | --- | --- |
|  | Frusemide | -0.16 | -0.46 – 0.13 | 0.29 |
|  | ACEI | 0.04 | -0.14 – 0.22 | 0.64 |
|  | Aspirin | -0.13 | -0.31 – 0.04 | 0.13 |
|  | IHD | 0.04 | -0.12 – 0.20 | 0.64 |
|  | Beta blocker | 0.05 | -0.14 – 0.24 | 0.60 |
|  | statin | -0.01 | -0.18 – 0.18 | 0.98 |

Model 1 was adjusted for smoking, DM, initial diameter and sex. Model 2 was adjusted for smoking, IHD, initial diameter, sex, DM, CCB, ACEI, frusemide, aspirin, BB and statin.

Abbreviations: AAA – abdominal aortic aneurysm, BB – beta blocker, CI – confidence interval, DBP – diastolic blood pressure, DM – diabetes mellitus, IHD – ischemic heart disease, N – sample size. Cited p values ≈ β = interaction of time and blood pressure groups.

# Table 3 – Association between DBP and AAA growth after removing outliers

|  | **Number of participants**  **(n = 1293,**  **number of observations = 6006)** | **Mean difference in AAA growth per year** | **95 % CI** | **P value** |
| --- | --- | --- | --- | --- |
| Unadjusted model | DBP ≤ 90 | REF |  |  |
|  | DBP > 90 | -1.81 | -2.47 - -1.15 | <0.001 |
| Adjusted model 1 | DBP ≤ 90 | REF |  |  |
|  | DBP > 90 | -0.02 | -0.15 – 0.10 | 0.72 |
|  | Initial diameter | 0.99 | 0.98 – 1.00 | <0.001 |
|  | Smoking | 0.06 | -0.06 – 0.18 | 0.30 |
|  | DM | -0.05 | -0.20 – 0.11 | 0.52 |
|  | Sex | -0.15 | -0.34 – 0.05 | 0.14 |
| Adjusted model 2 | DBP ≤ 90 | REF |  |  |
|  | DBP > 90 | -0.04 | -0.16 – 0.09 | 0.54 |
|  | Initial diameter | 0.99 | 0.98 – 1.00 | <0.001 |
|  | Smoking | 0.07 | -0.05 – 0.20 | 0.25 |
|  | DM | -0.05 | -0.20 – 0.10 | 0.53 |
|  | Sex | -0.13 | -0.33 – 0.06 | 0.18 |
|  | Frusemide | -0.18 | -0.39 – 0.04 | 0.10 |
|  | ACEI | 0.06 | -0.07 – 0.19 | 0.38 |
|  | Aspirin | -0.11 | -0.24 – 0.02 | 0.09 |
|  | IHD | -0.001 | -0.12 – 0.12 | 0.97 |
|  | Beta blocker | -0.03 | -0.18 – 0.12 | 0.63 |
|  | statin | -0.001 | -0.13 – 0.13 | 0.98 |

Model 1 was adjusted for smoking, DM, initial diameter and sex.

Model 2 was adjusted for smoking, IHD, initial diameter, sex, DM, CCB, ACEI, frusemide, aspirin, BB and statin. Cited p values ≈ β = interaction of time and blood pressure groups.

Abbreviations: BB – beta blocker, CI – confidence interval, DBP – diastolic blood pressure, DM – diabetes mellitus, IHD – ischemic heart disease, N – sample size.

#
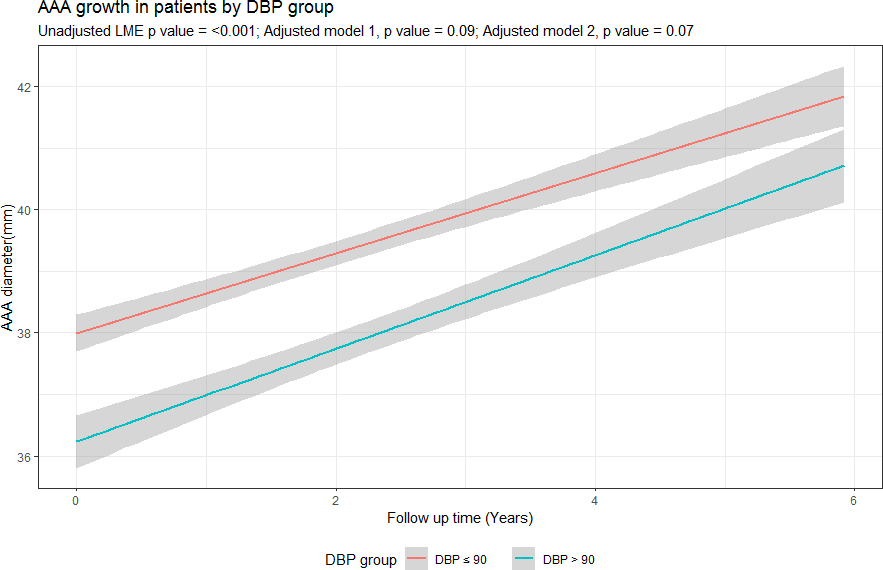
Figure 1: Association of diastolic blood pressure with abdominal aortic aneurysm growth.

The graph illustrates the mean (95% CI) AAA growth during follow up (years) according to diastolic blood pressure (DBP). The red line represents patients with DBP ≤ 90 mmHg and the green line represents patients with DBP >90 mmHg.

**Table 4 – Association between DBP (continuous variable) and AAA growth**

|  | **Number of participants (n = 1293, number of**  **observations = 6006)** | **Mean difference in AAA growth per year** | **95 % CI** | **P value** |
| --- | --- | --- | --- | --- |
| Unadjusted model | DBP | -0.07 | -0.09 - -0.04 | <0.001 |
| Adjusted model 1 | DBP | -0.004 | -0.01 – 0.002 | 0.21 |
| Adjusted model 2 | DBP | -0.004 | -0.01 – 0.002 | 0.16 |
| **After removing the outliers** | | | | |
| Unadjusted model | DBP | -0.07 | -0.09 - -0.05 | <0.001 |
| Adjusted model 1 | DBP | <0.001 | -0.005 – 0.003 | 0.69 |
| Adjusted model 2 | DBP | -0.002 | -0.007 – 0.002 | 0.32 |

Model 1 was adjusted for smoking, DM, initial diameter and sex.

Model 2 was adjusted for smoking, IHD, initial diameter, sex, DM, CCB, ACEI, frusemide, aspirin, BB and statin. Cited p values ≈ β = interaction of time and blood pressure groups.

Abbreviations: BB – beta blocker, CI – confidence interval, DBP – diastolic blood pressure, DM – diabetes mellitus, IHD – ischemic heart disease, N – sample size.
